# Critical role of leukotriene B_4_ receptor signaling in mouse 3T3-L1 preadipocyte differentiation

**DOI:** 10.1186/1476-511X-12-122

**Published:** 2013-08-09

**Authors:** Kae Hirata, Koichiro Wada, Yuka Murata, Atsushi Nakajima, Takashi Yamashiro, Yoshinori Kamisaki

**Affiliations:** 1 Department of Orthodontics and Dentofacial Orthopedics, Graduate School of Dentistry, Osaka University, Suita, Osaka 565-0871, Japan; 2 Department of Pharmacology, Graduate School of Dentistry, Osaka University, Suita, Osaka 565-0871, Japan; 3 Gastroenterology Division, Yokohama City University School of Medicine, Yokohama, Kanagawa 236-0004, Japan

**Keywords:** Leukotrienes, Preadipocyte differentiation, Mouse 3T3-L1 fibroblasts, BLT, siRNA

## Abstract

**Background:**

Various inflammatory mediators related to obesity might be closely related to insulin resistance. Leukotrienes (LTs) are involved in inflammatory reactions. However, there are few reports regarding the role of LTs in adipocyte differentiation. Therefore, we investigated the role of leukotriene B_4_ (LTB_4_)-leukotriene receptor (BLT) signaling in mouse 3T3-L1 fibroblastic preadipocyte differentiation to mature adipocytes.

**Methods:**

Mouse 3T3-L1 preadipocytes were treated with lipoxygenase (LOX) inhibitors, BLT antagonist, and small interfering RNA (siRNA) for BLT1 and BLT2 to block the LTB_4_-BLT signaling pathway, then the adipocyte differentiation such as lipid accumulation and the increase in triglyceride was evaluated.

**Results:**

Blockade of BLT signaling by treatment with a LOX inhibitor or a BLT antagonist suppressed preadipocyte differentiation into mature adipocytes. In addition, knockdown of BLT1 and BLT2 by siRNAs dramatically inhibited differentiation. These results indicate the LTB_4_-BLT signaling pathway may positively regulate preadipocyte differentiation and be a rate-limiting system to control adipocyte differentiation.

**Conclusions:**

The LTB_4_-BLT signaling pathway provides a potent regulatory signal that accelerates the differentiation of mouse 3T3-L1 preadipocytes. Further investigations are necessary to confirm the exact role of LTB_4_ and BLTs signaling pathways in preadipocyte differentiation.

## Background

Diabetes mellitus, hyperlipidemia, hypertension, and atherosclerosis have recently been defined as typical life style-related diseases. A common background of these diseases is obesity, which is thought to cause insulin resistance resulting in the onset of disease [1,2]. Recently, the incidence of obesity and associated metabolic syndrome has dramatically increased. Although high caloric western-style foods are believed to be the main cause of this dramatic increase, other possible risk factors could exist. The involvement of various inflammatory mediators such as tumor necrosis factor alpha (TNFα) and interleukin 6 (IL-6) on obesity might be closely related to insulin resistance [1-4]. One of the most important organs in obesity and insulin resistance are the adipose tissues, as adipocytes generate adipocytokines that are important in the onset of metabolic syndrome [1,2,5].

Leukotrienes (LTs) such as leukotriene B_4_, C_4_, and D_4_ (LTB_4_, LTC_4_, and LTD_4_, respectively) are generated through lipoxygenase (LOX) pathways and induce inflammatory and allergic reactions such as leukocyte activation, capillary permeability, and bronchial contraction [6,7]. LTB_4_ binds to specific receptors, BLT1 and BLT2, to activate signaling pathways [8,9]. LTs have been reported to be involved in the proliferation of epithelial, endothelial and mesangial cells [10,11]. In addition, we previously reported that LTB_4_ controls immature neural stem cell proliferation and differentiation via the BLT signaling pathway [12]. Thus, LTB_4_ and its signaling pathway might be involved in cell proliferation and differentiation. However, there have been few reports regarding the role of LTs in adipocyte differentiation.

In this study, we investigated the role of LTB_4_ and BLTs signaling pathways in preadipocyte differentiation in a mouse fibroblastic 3T3-L1 cell line, a widely used cell line for research of preadipocyte differentiation [13]. We analyzed the effects of LOX inhibitors, a BLT antagonist and BLTs-specific siRNAs on the differentiation of 3T3-L1 to clarify the function of BLTs on preadipocyte differentiation. Our results suggest a potentially important and novel role for LTB_4_ and BLT functions on preadipocyte differentiation.

## Results

### Effects of LOX inhibitors and BLT antagonist on mouse 3T3-L1 preadipocyte differentiation

**Figure 1:**
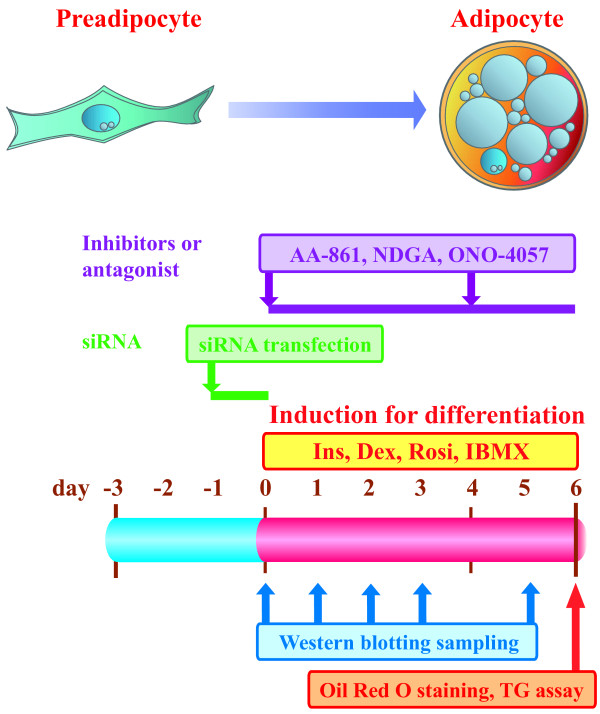
Schematic protocol for mouse 3T3-L1 preadipocyte differentiation.

**Figure 2:**
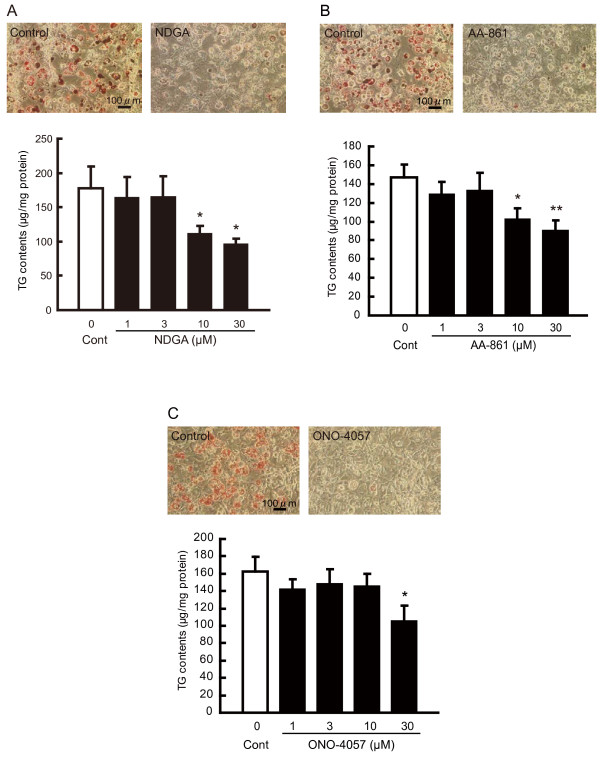
**Effects of LOX inhibitors and the BLT antagonist on mouse 3T3-L1 preadipocyte differentiation. (A)**, **(B)** and **(C)**: Effects of NDGA **(**LOX inhibitor, **A)**, AA-861 **(**5-LOX inhibitor, **B)**, or ONO-4057 **(**a specific BLT antagonist, **C)** on lipid accumulation in mouse 3T3-L1 preadipocytes. Mouse 3T3-L1 preadipocytes were treated with NDGA, AA-861 or ONO-4057 for 6 days. Then, accumulation of triacylglycerol (TG), a marker of lipid accumulation (bottom panel), in matured adipocytes was measured and expressed as TG contents (μg/mg protein). Each column represents the mean ± SEM from 4-8 independent experiments. *P<0.05, **P<0.01 vs. vehicle control. Upper left panel shows representative photographs of differentiated mouse 3T3-L1 preadipocytes treated with vehicle for 6 days. Upper right panel shows representative photographs of undifferentiated mouse 3T3-L1 preadipocytes treated with NDGA, AA-861 or ONO-4057 for 6 days. Cells were stained with Oil Red O method to visualize lipid accumulation. Scale bar represents 100 μm.

Mouse 3T3-L1 cells can differentiate from fibroblastic cells into mature adipocytes in induction medium for differentiation containing insulin (INS), dexamethasone (DEX), 3-isobutyl-1-methylxanthine (IBMX) and rosiglitazone (ROSI), a specific ligand for peroxisome proliferator-activated receptor gamma (PPARγ) [13,14]. We used these induction conditions for the differentiation of mouse 3T3-L1 preadipocytes (Figure 1). Nordihydroguaiaretic acid (NDGA), a pan-LOX inhibitor, inhibited the accumulation of lipids, the decrease of triacylglycerol (TG) contents and the index of mouse 3T3-L1 preadipocyte differentiation into mature adipocytes (Figure 2A). No alterations in cell proliferation were observed under our experimental conditions (data not shown). AA-861, a 5-LOX inhibitor, also inhibited the differentiation of mouse 3T3-L1 preadipocytes into mature adipocytes (Figure 2B). These suppressive effects occurred in a concentration-dependent manner. In addition, ONO-4057, a specific LTB_4_ receptor antagonist, suppressed mouse 3T3-L1 preadipocyte differentiation (Figure 2C). These results suggest that the LTB_4_-BLT signaling pathway may be involved in preadipocyte differentiation.

### Effect of BLT1 and BLT2 knockdown by siRNA on mouse 3T3-L1 preadipocyte differentiation

**Figure 3:**
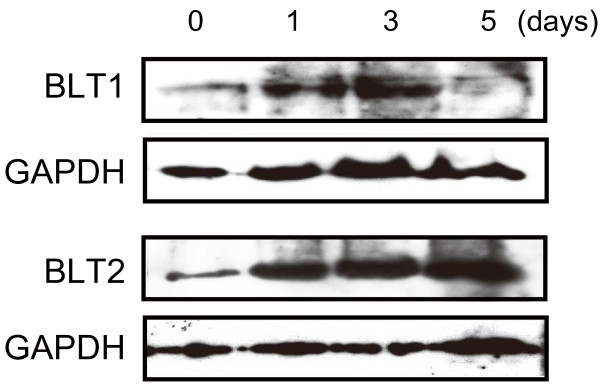
**Expression of BLT1 and BLT2 during preadipocyte differentiation.** Detection of BLT1 and BLT2 was performed by western blot analysis using anti-BLT1 or -BLT2 antibodies. GAPDH was used as an internal standard for confirmation of equally applied amounts of protein.

To investigate whether BLTs are expressed on preadipocytes, we performed western blot analysis, which showed that both BLT1 and BLT2 were expressed in mouse 3T3-L1 preadipocytes from the start to late phases of differentiation (Figure 3). In addition, the level of LTB_4_ secreted from preadipocytes into the culture medium was 31.8 ± 8.4 nmol/L (mean ± SEM, n=3). These results indicate that the BLT-signaling pathway induces the differentiation of preadipocytes to mature adipocytes.

**Figure 4:**
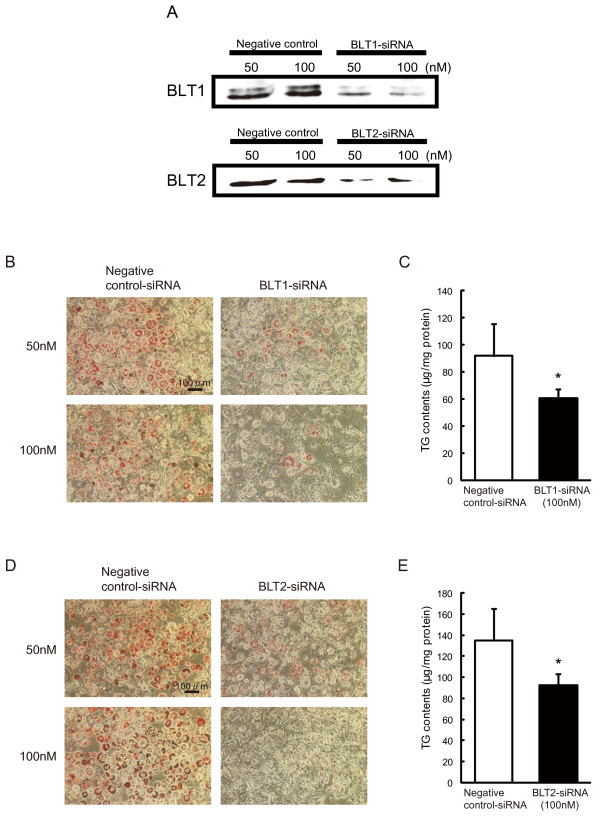
**Effect of BLT1 and BLT2 knockdown by siRNA on mouse 3T3-L1 preadipocyte differentiation. (A)**: Confirmation of BLT1 and BLT2 knockdown by specific siRNAs. Negative control is cells treated with negative control siRNA (Stealth RNAi Negative Control Duplexes). **(B)** and **(C)**: Effect of BLT1 knockdown by siRNA. Representative microscopic **(B)** images of differentiated mouse 3T3-L1 adipocytes by Oil Red O staining. Scale bar represents 100 μm. **(C)**: Accumulation of TG in mature adipocytes was measured and expressed as TG contents (μg/mg protein). Each column represents the mean ± SEM from 3-5 independent experiments. *P<0.05 vs. negative control-siRNA treatment. **(D)** and **(E)**: Effect of BLT2 knockdown by siRNA. Representative microscopic **(D)** images of differentiated mouse 3T3-L1 adipocytes by Oil Red O staining. Scale bar represents 100 μm. **(E)**: Accumulation of TG in mature adipocytes was measured and expressed as TG contents (μg/mg protein).

Therefore, we designed siRNAs specific for BLT1 or BLT2 to knockdown receptor expression. siRNAs against BLT1 and BLT2 successfully suppressed the expression of BLT1 and BLT2 (Figure 4A). Indicators of preadipocyte differentiation such as lipid accumulation and TG contents were decreased by BLT1 siRNA (Figure 4B and C). Similar results were observed with BLT2 siRNA (Figure 4D and E). These results clearly indicated that the LTB_4_-BLT signaling pathway accelerates mouse 3T3-L1 preadipocyte differentiation, and blockade or knockdown of BLTs leads to the suppression of preadipocyte differentiation.

### Combination knockdown of BLT1 and BLT2 by siRNA on mouse 3T3-L1 preadipocyte differentiation

**Figure 5:**
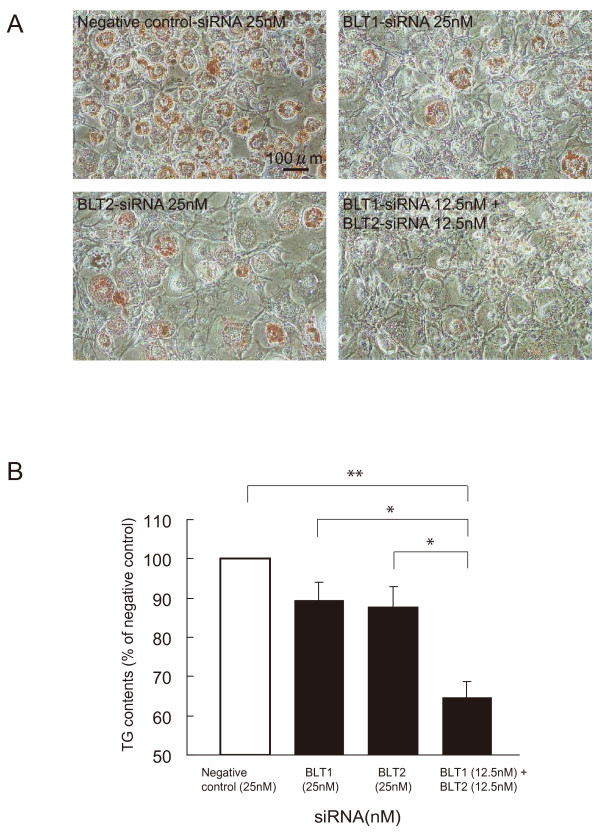
**Combination knockdown of BLT1 and BLT2 by siRNA on mouse 3T3-L1 preadipocyte differentiation.** Mouse 3T3-L1 preadipocytes were treated with a combination of BLT1-siRNA (12.5 nM) and BLT2-siRNA (12.5 nM). **(A)**: Representative microscopic images of differentiated mouse 3T3-L1 adipocytes by Oil Red O staining. Scale bar represents 100 μm. **(B)**: Accumulation of TG in mature adipocytes was measured and expressed as TG contents (% of negative control). Each column represents the mean ± SEM from 3 independent experiments. *P<0.05, **P<0.01 vs. negative control-siRNA treatment.

To clarify the role of each receptor, BLT1 and BLT2, on adipocyte differentiation, we performed combination treatment of BLT1-siRNA and BLT2-siRNA. The combined treatment of BLT1-siRNA (12.5 nM) and BLT2-siRNA (12.5 nM) remarkably decreased lipid accumulation and TG contents in comparison to single knockdown (Figure 5A and B) indicating that combination knockdown of BLT1 and BLT2 by specific siRNA efficiently suppressed preadipocyte differentiation.

## Discussion

The mouse fibroblastic 3T3-L1 cell line established by Green is widely used for the investigation of adipocyte differentiation [13,15,16]. The involvement of various molecules for adipocyte differentiation has been investigated using this cell line. However, it is not fully understood whether inflammation-related lipid mediators, such as LTs and prostaglandins (PGs), promote or inhibit the onset of metabolic syndrome. Several previous reports indicated that PGD_2_-derived 15-deoxy-Δ^12,14^-PGJ_2_ promoted adipocyte differentiation via activation of the PPARγ pathway [17,18]. Therefore, cyclooxygenase-related prostanoids are considered involved in the enhancement of adipocyte differentiation. In contrast, there have been few reports regarding the involvement of LOX-related metabolites in adipocyte differentiation.

In this study, 5-LOX inhibitors and a specific LTB_4_ receptor antagonist inhibited the differentiation of mouse 3T3-L1 preadipocytes into mature adipocytes. Furthermore, BLT1 and BLT2 knockdown by siRNA suppressed mouse 3T3-L1 preadipocyte differentiation. In addition, combination knockdown of BLT1 and BLT2 by siRNA on mouse 3T3-L1 preadipocytes remarkably decreased lipid accumulation and TG contents in comparison to single knockdown alone. These results clearly indicate that the LTB_4_-BLT signaling pathway is involved in mouse 3T3-L1 preadipocyte differentiation, and blockade or knockdown of BLTs leads to the suppression of preadipocyte differentiation.

Furthermore, we demonstrated that both LTB_4_ receptors, BLT1 and BLT2, were expressed in mouse 3T3-L1 preadipocytes. We also confirmed the release of LTB_4_ from preadipocytes into the culture medium. These results indicate that a paracrine or autocrine pathway of BLT-signaling operates in preadipocytes, for the positive regulation of mouse 3T3-L1 preadipocyte differentiation from adipocyte progenitors, because inhibition of this pathway with LOX inhibitors, a BLT antagonist, or siRNAs for BLTs induced the inhibition of preadipocyte differentiation.

Interestingly, a recent paper showed that deletion of BLT1 protected mice from high-fat diet-induced insulin resistance [19]. Such observations clearly show that BLT1 signaling is closely involved in insulin resistance. However, the results of a BLT1 knockout mouse study were considered to be due to systemic mechanisms. Therefore, the local action of BLT1 signaling on adipose tissues should be investigated. Our present data using 3T3-L1 adipocytes may partly support these previous observations. To clarify the issue, an adipocyte specific BLT1-conditional knockout mouse study is required. The involvement of BLT1 signaling may be important for adipocyte differentiation and related systemic disorders such as insulin resistance and obesity.

To investigate the potential mechanisms of BLT signaling-mediated acceleration of 3T3-L1 preadipocyte differentiation, we performed DNA microarray analysis to identify the molecules regulated by LTB_4_-BLT signaling. Many molecules were significantly altered by treatment with a 5-LOX inhibitor, AA-861 or a specific BLT antagonist, ONO-4057 (unpublished data). Among them, we initially focused on the expressions of PPARγ and CCAAT-enhancer-binding protein, alpha (C/EBPα) which is known as important key transcriptional regulators to control adipocyte differentiation. However, both molecules did not show significant changes at the microarray analysis. Namely, increase in PPARγ expression was from 0.9 to 1.3-fold, that in C/EBPα expression was from 0.7 to 1.1-fold, respectively. Therefore, it is expected that LTB_4_-BLT signaling pathway promoted 3T3-L1 preadipocyte differentiation via other molecules independent to PPARγ or C/EBPα. Further investigations will be required to clarify the molecules.

In conclusion, the LTB_4_-BLT signaling pathway provides a potent regulatory signal that accelerates the differentiation of mouse 3T3-L1 preadipocytes. Our results imply a potentially important and novel role for LTB_4_ and BLT functions on preadipocyte differentiation. Further investigations are necessary to confirm the exact role of LTB_4_ and BLTs signaling pathways in preadipocyte differentiation.

## Material and methods

### Reagents and antibodies

INS, DEX and IBMX were purchased from Sigma Japan (Tokyo, Japan). ROSI was purchased from GlaxoSmithKline K. K. (Tokyo, Japan). LT synthetase, LOX inhibitor, NDGA, and 5-LOX specific inhibitor, AA-861, were purchased from Sigma Japan. ONO-4057, a specific BLT antagonist, was a kind gift from ONO Pharmaceutical Co. Ltd. (Osaka, Japan). Anti-BLT1 and -BLT2 polyclonal antibodies were purchased from Cayman Chemicals (Ann Arbor, MI, USA).

### Cell culture and induction of adipocyte differentiation

Mouse 3T3-L1 preadipocytes have been frequently used to study the differentiation of preadipocytes in vitro. Cell culture and induction of differentiation of preadipocytes were performed according to the previously described method (Figure 1) [14,20]. Briefly, mouse 3T3-L1 preadipocytes were cultured in Dulbecco’s modified Eagle’s medium (DMEM, Sigma Japan) supplemented with 10% fetal bovine serum, 1% MEM non-essential amino acids, 100 IU/mL penicillin and 0.1 mg/mL streptomycin. At 3 days after reaching confluency (day 0), the medium was replaced with induction medium for differentiation containing INS (150 nM), DEX (1 μM), IBMX (100 μM) and ROSI (PPARγ-ligand, 1 μM). The differentiation medium was changed every 4 days until analysis (day 6).

The LOX inhibitor (NDGA/AA-861) or specific BLT antagonist (ONO-4057) was prepared in dimethyl sulfoxide (DMSO, Sigma Japan) and added to the differentiation medium from day 0 to day 6 (Figure 1). The DMSO concentration was maintained up to 0.1% of the total volume, and preliminary experiments demonstrated no significant effects of 0.1% DMSO on cell differentiation.

### Evaluation of adipocyte differentiation

Differentiation of preadipocytes to mature adipocytes was visually monitored by microscopic observation after Oil red O staining [14,15]. In addition, the amount of triglyceride, an index of lipid accumulation, was quantitatively measured using a Triglyceride E-test Wako kit (Wako Pure Chemicals, Tokyo, Japan). The amount of triglyceride was normalized by protein amount and expressed as TG contents (μg/mg protein).

### siRNA for knockdown of BLT1 and BLT2

We designed small interfering RNA (siRNA) for knockdown of BLT1 and BLT2 using an siRNA system (Qiagen, Tokyo, Japan). The sequences of the sense and antisense strand for BLT1 used were 5’-CAACCUACACUUCCUAUUA-3’, and 5’-UAAUAGGAAGUGUAGGUUG-3’, respectively. The sequences of the sense and antisense strand for BLT2 used were 5’-GGGACUUAACAUACUCUUA-3’, and 5’-UAAGAGUAUGUUAAGUCCG-3’, respectively.

For transfection, siRNAs or negative control siRNA (Stealth RNAi Negative Control Duplexes, Invitrogen, Tokyo, Japan) were combined with Lipofectamine RNAiMAX (Invitrogen) and incubated for 20 minutes at room temperature to produce the transfection mixture. Then, the transfection mixture was added to preadipocytes at a final concentration of 25, 50 and 100 nM siRNA (Figure 1). At 24 hours after the start of transfection, the medium was replaced with differentiation medium to induce differentiation. Samples were collected at days 1, 2, 3 and 5 day for western blot analysis, and at day 6 for TG assay and Oil red O staining.

### Statistical analysis

Results were expressed as the mean ± SEM. Statistical comparisons were performed using the Student’s *t*-test or Tukey’s method after analysis of variance (ANOVA). The results were considered significantly different at *P* <0.05.

## Abbreviations

LT: Leukotriene; LOX: Lipoxygenase; siRNA: Small interfering RNA; TNFα: Tumor necrosis factor alpha; IL-6: Interleukin 6; INS: Insulin; DEX: Dexamethasone; IBMX: 3-Isobutyl-methylxanthine; ROSI: Rosiglitazone; PPARγ: Peroxisome proliferator-activated receptor gamma; NDGA: Nordihydroguaiaretic acid; TG: Triacylglycerol; PG: Prostaglandin; C/EBPα: CCAAT-enhancer-binding protein, alpha; DMEM: Dulbecco’s modified Eagle’s medium; DMSO: Dimethyl sulfoxide; ANOVA: Analysis of variance.

## Competing interests

The authors declare that they have no competing interests.

## Authors’ contributions

KH performed all experiments and statistical analysis, discussion of results and drafted the manuscript. KW conceived the study, participated in discussion of the results, provided additional funding for the study. YM assisted in performance of some experiments. AN, YK, TY participated in discussion of the results. All authors read and approved the final manuscript.
